# A Case of SLE Flare With Class IV Lupus Nephritis and Tuberculous Meningitis Presenting as Neuropsychiatric Symptoms: A Diagnostic Dilemma

**DOI:** 10.1155/crrh/8150858

**Published:** 2026-11-02

**Authors:** Govinda Bhandari, Anupam Katuwal Chhetri, Rajan Gyawali, Binay Chapagain, Anush Acharya

**Affiliations:** ^1^ Institute of Medicine Maharajgunj Medical Campus Kathmandu Nepal; ^2^ Department of Internal Medicine Tribhuvan University Teaching Hospital Kathmandu Nepal teachinghospital.org.np

## Abstract

**Background:**

Systemic lupus erythematosus (SLE) is a multisystem autoimmune disease with potential neuropsychiatric involvement (NPSLE) and increased susceptibility to opportunistic infections like tuberculosis (TB), particularly in endemic regions such as Nepal where the estimated incidence is 229 per 100,000 population. Differentiating NPSLE from tuberculous meningitis (TBM) is diagnostically challenging due to overlapping clinical and cerebrospinal fluid (CSF) findings.

**Case Presentation:**

A 25‐year‐old male with a 1‐year history of SLE presented with facial puffiness, oral ulcers, polyarthritis, lower limb edema, and a 6‐week history of renal symptoms. Laboratory investigations revealed microcytic anemia (Hb 7.4 g/dL), nephrotic‐range proteinuria (urine protein‐to‐creatinine ratio 4.8 g/g), strongly positive anti‐dsDNA, and low complement levels. ENA panel was positive for anti‐Ro/SSA but negative for antiribosomal P and antiphospholipid antibodies. Renal biopsy confirmed class IV lupus nephritis (Activity Index 11/24 and Chronicity Index 3/12). Following intravenous methylprednisolone pulse therapy (500 mg/day for 3 days), he developed generalized tonic‐clonic seizures and altered sensorium. EEG revealed epileptiform discharges. CSF analysis showed lymphocytic pleocytosis (500 cells/μL and 80% lymphocytes), elevated protein (578 mg/dL), low glucose (1.4 mmol/L), and elevated ADA (10.9 U/L), suggestive of TBM despite negative GeneXpert MTB/RIF, viral PCR, and cryptococcal antigen assays. MRI brain showed cerebral atrophy, calcified granulomas in the right temporal lobe, and a nonspecific demyelinating white matter lesion. Based on clinical, CSF, and imaging findings, empirical antitubercular therapy (ATT) was initiated alongside tailored immunosuppression. The patient’s neurological and renal status markedly improved with complete resolution of seizures.

**Discussion:**

Distinguishing NPSLE from CNS infections requires careful evaluation of serial CSF parameters, biomarkers, and MRI findings. In endemic regions, empirical ATT should be initiated early when clinical and laboratory features suggest TBM, even when molecular or culture tests are negative.

**Conclusion:**

In SLE patients with neuropsychiatric symptoms in TB‐endemic areas, clinicians must consider TBM even when definitive microbiological tests are negative. Early empirical treatment can significantly improve patient survival and long‐term outcomes.

## 1. Introduction

Systemic lupus erythematosus (SLE) is a chronic, multisystem autoimmune disease characterized by autoantibody production and immune complex deposition, affecting various organs including the central nervous system (CNS) [1]. Neuropsychiatric manifestations (NPSLE) encompass a spectrum of neurological and psychiatric symptoms, with reported prevalence rates ranging from 37% to 95% depending on diagnostic criteria and cohorts [1, 2]. Among the renal complications, class IV diffuse proliferative lupus nephritis (LN) carries the poorest long‐term renal outcomes [3].

Patients with SLE are highly susceptible to opportunistic infections due to baseline immune dysregulation and therapeutic immunosuppression [4, 5]. In endemic countries such as Nepal (annual TB incidence ∼229 per 100,000 population) [6], *Mycobacterium tuberculosis* is a major infectious threat [4, 7]. CNS infections in SLE present diagnostic dilemmas because their clinical and cerebrospinal fluid (CSF) profiles mimic NPSLE [8]. In addition to *M. tuberculosis*, other opportunistic pathogens causing infectious meningitis in SLE patients include *Cryptococcus neoformans*, cytomegalovirus (CMV), Epstein–Barr virus (EBV), and herpes simplex virus (HSV) [8]. Establishing a prompt diagnosis requires CSF analysis (cell count, differential, protein, glucose, ADA, GeneXpert, India ink, cryptococcal antigen, and viral PCR panels) alongside neuroimaging [9–12].

This case report details a 25‐year‐old male with active SLE who developed concurrent class IV LN and tuberculous meningitis (TBM) presenting as acute neuropsychiatric collapse, highlighting the diagnostic considerations and therapeutic balance between immunosuppression and antimicrobial therapy [7, 13, 14].

## 2. Case Presentation

A 25‐year‐old male with a known diagnosis of SLE for 1 year presented to the outpatient department of a university teaching hospital with progressive bilateral lower limb swelling for 6 weeks and polyarthralgia involving bilateral wrists, knees, and elbows for 1 month. Due to financial constraints associated with out‐of‐pocket costs for long‐term medications in our setting, he reported poor adherence to prescribed treatments over the preceding 6 months, leading to disease flare. At initial diagnosis, he had hematological and mucocutaneous manifestations and was treated with hydroxychloroquine 200 mg once daily, mycophenolate mofetil 1 g twice daily, and oral prednisolone 1 mg/kg/day (50 mg daily).

On admission, he complained of generalized weakness, myalgia, active painful oral ulcers, malar rash, and facial puffiness. Regarding negative review of systems, there were no symptoms of systemic or local infection such as fever, cough, shortness of breath, dysuria, chest pain, or night sweats; similarly, there were no baseline focal neurological deficits, morning stiffness, or altered sensorium.

Initial laboratory investigations revealed severe renal impairment (serum urea 25 mmol/L and creatinine 291 μmol/L) and electrolyte imbalance (sodium 134 mmol/L and potassium 5.6 mmol/L). Hematological analysis demonstrated microcytic hypochromic anemia with hemoglobin 7.4 g/dL, MCV 67 fL, MCH 23 pg, serum iron 24 μg/dL, and total iron‐binding capacity 137 μg/dL (Table 1). Lipid profiling revealed hypercholesterolemia (total cholesterol 9.0 mmol/L, triglycerides 6.4 mmol/L, and LDL 6.3 mmol/L). Urinalysis demonstrated heavy proteinuria with a spot urine protein‐to‐creatinine ratio (UPCR) of 4.8 g/g, consistent with nephrotic‐range proteinuria. Serum albumin was 18 g/L, total protein was 42 g/L, and muscle CPK was 740 IU/L. Intact parathyroid hormone (iPTH) was markedly elevated at 289.2 pg/mL, representing secondary hyperparathyroidism secondary to acute‐on‐chronic renal impairment and altered calcium‐phosphate‐vitamin D metabolism. Immunological workup showed strongly positive anti‐dsDNA (150 IU/mL), hypocomplementemia (C3 45 mg/dL and C4 8 mg/dL), and positive anti‐Ro/SSA antibodies. Anti‐La/SSB, anti‐Sm, anti‐RNP, antiribosomal P, and antiphospholipid antibodies (lupus anticoagulant, anticardiolipin, and anti‐beta‐2‐glycoprotein I) were negative.

**TABLE 1:** Serial laboratory investigations and disease biomarkers.

Parameter (reference range)	Admission	Peak deterioration	Discharge
Hemoglobin (13.0–17.0 g/dL)	7.4	6.8	10.2
MCV (80–100 fL)	67	68	78
Leukocyte count (4000–11,000/μL)	4200	11,500	6800
Platelet count (150–450 × 10^3^/μL)	110	145	210
Serum urea (2.5–7.1 mmol/L)	25.0	38.2	8.1
Serum creatinine (62–106 μmol/L)	291	480	118
Serum sodium (135–145 mmol/L)	134	131	138
Serum potassium (3.5–5.0 mmol/L)	5.6	5.1	4.2
Total cholesterol (< 5.2 mmol/L)	9.0	—	5.1
Triglycerides (< 1.7 mmol/L)	6.4	—	2.2
Serum albumin (35–50 g/L)	18	16	31
Urine protein‐to‐creatinine ratio (UPCR; < 0.2 g/g)	4.8	—	0.9
Serum intact PTH (15–65 pg/mL)	289.2	—	72.0
Anti‐dsDNA (< 30 IU/mL)	150	—	42
Complement C3 (90–180 mg/dL)	45	—	78
Complement C4 (10–40 mg/dL)	8	—	18

Due to symptomatic severe anemia (fatigue and resting tachycardia) with acute renal crisis, the patient was transfused with 2 units of packed red blood cells (PRBCs) to achieve rapid hemodynamic and tissue oxygenation stability prior to initiating long‐term erythropoiesis‐stimulating agents and iron therapy. Renal biopsy confirmed class IV diffuse proliferative LN with an Activity Index of 11/24 and Chronicity Index of 3/12 [3]. He was started on intravenous methylprednisolone pulse therapy at 500 mg daily for 3 consecutive days, followed by oral prednisolone 1 mg/kg/day.

On hospital Day 6, the patient developed high‐grade fever, severe headache, two episodes of generalized tonic‐clonic seizures (GTCS), and altered sensorium. He was transferred to the intensive care unit (ICU) and intubated for airway protection. Seizures were managed with intravenous midazolam, levetiracetam, lacosamide, and sodium valproate. Electroencephalography (EEG) showed generalized interictal epileptiform discharges with sharp‐and‐spike wave activity and background slowing, indicating grade III encephalopathy.

A lumbar puncture yielded turbulent CSF with elevated opening pressure (240 mmH2O). CSF analysis showed a total nucleated cell count of 500 cells/μL (80% lymphocytes and 20% neutrophils), elevated protein (578 mg/dL), markedly decreased glucose (1.4 mmol/L; simultaneous blood glucose 5.8 mmol/L), and elevated adenosine deaminase (ADA) of 10.9 U/L. Gram stain, Ziehl–Neelsen stain, GeneXpert MTB/RIF, HSV‐1/2 PCR, India ink, and cryptococcal antigen lateral flow assay were all negative. Sputum and bronchoalveolar lavage (BAL) cultures for *M. tuberculosis* were negative, and chest imaging was normal. Brain MRI demonstrated global cerebral atrophy, blooming foci in the right temporal lobe consistent with calcified granulomas, and a nonspecific subcortical T2/FLAIR hyperintense demyelinating focus (Figure 1).

**FIGURE 1:**
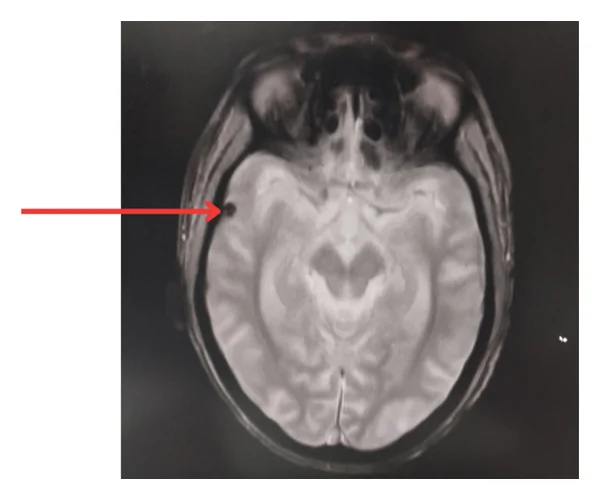
Blooming foci in the right temporal lobe consistent with calcified granulomas and global cerebral atrophy.

Despite negative GeneXpert results, the combination of marked hypoglycorrhachia, high protein, lymphocytic pleocytosis, elevated ADA, and temporal lobe granulomas raised strong suspicion of TBM. Empirical antitubercular therapy (ATT) consisting of isoniazid (300 mg/day), rifampicin (600 mg/day), pyrazinamide (1500 mg/day), ethambutol (1200 mg/day), and pyridoxine (50 mg/day) was initiated alongside stress‐dose corticosteroids.

During the ICU stay, transient worsening of respiratory status required temporary mechanical ventilation, and transient deterioration of renal function was managed with hemodialysis sessions. Following initiation of ATT, the patient’s neurological status progressively improved; seizures ceased, consciousness normalized, and he was successfully extubated [15]. Repeat CSF analysis at 3 weeks demonstrated clearing pleocytosis and normalizing glucose. Subsequent 6‐week CSF mycobacterial cultures yielded no growth, consistent with the low yield of paucibacillary CSF cultures in TBM. Oral mycophenolate mofetil (MMF) was safely reintroduced at a maintenance dose of 1.5 g/day for LN. The patient was discharged after 4 weeks of hospitalization in stable condition and remains in regular outpatient follow‐up with sustained recovery.

## 3. Discussion

This case illustrates the diagnostic and therapeutic challenge of differentiating NPSLE from TBM in an immunocompromised patient with active LN [4, 7, 13, 14]. Both conditions can present with fever, headache, altered mentation, seizures, and focal neurological signs [1, 16–18]. Differentiating autoimmune CNS flare from severe infection is paramount, as misdiagnosis can lead to inappropriate escalation of immunosuppression, resulting in fatal infectious outcomes [7, 8].

The 1999 American College of Rheumatology (ACR) classification defines 19 neuropsychiatric syndromes associated with SLE, divided into 12 CNS syndromes (aseptic meningitis, cerebrovascular disease, demyelinating syndrome, headache, movement disorder, myelopathy, seizure disorders, acute confusional state, anxiety disorder, cognitive dysfunction, mood disorder, and psychosis) and 7 peripheral nervous system syndromes (acute inflammatory demyelinating polyradiculoneuropathy, autonomic neuropathy, mononeuropathy, myasthenia gravis, cranial neuropathy, plexopathy, and polyneuropathy) [17, 18]. Although positive antiribosomal P and antiphospholipid antibodies are associated with an increased risk of NPSLE, their absence in our patient did not exclude autoimmune CNS activity; however, the presence of marked hypoglycorrhachia strongly favored an infectious etiology [8–10].

In SLE patients with neuropsychiatric symptoms, differentiating infectious meningitis from NPSLE requires careful interpretation of clinical and biomarker profiles [8]. Infectious meningitis typically presents with high fever, severe headache, markedly elevated CRP, and recent high‐dose corticosteroid therapy, whereas NPSLE flares more often occur in the absence of fever or high CRP [8]. Common infectious etiologies in SLE patients include *M. tuberculosis* and *Cryptococcus neoformans*, the latter being the second most common cause of fungal CNS infection in lupus patients [5, 7].

CSF ADA serves as a valuable surrogate marker for TBM, with sensitivity around 85% and specificity around 90% at cutoffs of 8–10 U/L [9, 10, 15]. However, clinicians must be aware of false‐positive CSF ADA elevations, which can occur in purulent bacterial meningitis, CNS lymphoma, neurosarcoidosis, and cerebral toxoplasmosis [9, 15]. GeneXpert MTB/RIF demonstrates high specificity (> 98%) but modest sensitivity (50%–60%) in CSF due to the paucibacillary nature of TBM [11]. Thus, a negative GeneXpert or culture result does not rule out TBM [11]. While subacute TBM typically presents with lymphocytic pleocytosis, acute early stages may manifest with transient neutrophilic predominance; recognizing this temporal shift avoids misdiagnosing early TBM as acute pyogenic bacterial meningitis [9, 10].

Neuroimaging features differ between NPSLE and TBM [8, 12]. Brain MRI in TBM most frequently demonstrates hydrocephalus (present in approximately 80% of cases), basal leptomeningeal enhancement, ischemic brain infarctions, and tuberculomas [9, 15]. In contrast, NPSLE classically presents with subcortical white matter hyperintensities, cerebral atrophy, or microvascular infarctions [12]. In our patient, MRI demonstrated cerebral atrophy, a nonspecific white matter hyperintensity, and calcified temporal lobe granulomas [12]. Calcified granulomas represent prior exposure or healed tuberculomas rather than pathognomonic active TBM, but in the clinical context of lymphocytic pleocytosis and elevated CSF ADA, they supported the diagnosis of TB involvement [9, 12, 15].

Managing concurrent class IV LN and TBM requires balancing effective antimicrobial treatment against essential immunosuppression [3, 7]. Pulsed corticosteroids administered for severe LN can exacerbate uncontrolled infection, yet withholding immunosuppression risks irreversible renal damage [3, 7]. In our patient, initiation of empirical ATT combined with controlled corticosteroids allowed clinical recovery, after which MMF was safely reintroduced for ongoing LN management [7, 15].

## 4. Conclusion

This case demonstrates that in SLE patients presenting with neuropsychiatric symptoms in TB‐endemic areas, clinicians must maintain a high index of suspicion for TBM [7, 13, 14]. Because diagnostic tests such as GeneXpert and mycobacterial culture have limited sensitivity in CSF, empirical ATT should be initiated early when clinical, laboratory, and imaging features support the diagnosis [11, 15]. Early empirical intervention combined with balanced immunosuppression can prevent mortality and achieve favorable clinical recovery [7, 15].

## Funding

No funding was received for this manuscript.

## Ethics Statement

Institutional Review Board (IRB) exemption was obtained from the local ethics authority. Written informed consent was obtained from the patient for publication of this case report and associated clinical images.

## Conflicts of Interest

The authors declare no conflicts of interest.

## Data Availability

The data that support the findings of this study are available on request from the corresponding author. The data are not publicly available due to privacy or ethical restrictions.
